# 

**Published:** 1975-04

**Authors:** 


The
Bristol Medico-Chirurgical Journal
(Incorporating the Medical Journal of the South-West)
Established 1883

Vol. 90 (ii) APRIL 1975 No. 334

### BRISTOL MEDICO-CHIRURGICAL JOURNAL

EDITOR: D. S. REEVES, M.B., M.R.C.Path.

Published Quarterly

Annual Subscription £2 Post Free

### BRISTOL MEDICO-CHIRURGICAL SOCIETY

**President 1974/75:** Dr. J. Apley, O.B.E.

**President-elect:** D. C. Bodenham, Esq.

**Honorary Secretary:** A. J. Webb, Esq., 7 Percival Road, Bristol 8.

**Honorary Treasurer:** Dr. Frank Ross, Bristol Royal Infirmary, Bristol 2.

The Society was founded in 1874. In 1883 publication of the Journal began and has continued quarterly ever since then. During the years 1949 to 1962 it appeared under the title of "The Medical Journal of the South West".

### THE BRISTOL MEDICO-CHIRURGICAL JOURNAL

**Editorial Committee**

Editor Dr. D. S. Reeves, Southmead Hospital, Bristol.

Members Dr. Rhys Davies.
Dr. M. B. Lennard.
Professor R. C. Wofinden.
Dr. D. W. Wright.
The Hon. Treasurer and Hon. Secretary of the Society.

Secretary Mr. B. P. Jones, The Library, Medical School, University Walk, Bristol BS8 1TD.

### NOTICE TO CONTRIBUTORS

The Journal is especially concerned to provide a place for the recording of medical thought, interests, and practice in and around Bristol. It therefore welcomes articles that, as well as being of immediate interest to members, will document the local and contemporary medical scene for our successors.

Original articles are invited provided that they have not been published elsewhere. References in the text should be by author's name, with year of publication in parenthesis; they should be listed at the end in alphabetical order, the name of each journal or book being given in full—not abbreviated—and the title of the article added. Illustrations should be supplied ready for reproduction. Line drawings should be in Indian ink on firm white paper or board. The author will be informed if it is found necessary to ask him to pay for the making of blocks.

The following contributions will be especially welcome; notes of forthcoming events, meetings held, degrees conferred, staff appointments, honours and public appointments and other local news.

### University of Bristol

### Examinations Results and Dissertations Approved

**FINAL EXAMINATION FOR THE DEGREE OF B.D.S. (SECTION I) DECEMBER 1974**

**PASS**

Catherine ASHER
Paul Robert BAINES
Ramon BEDI
Glen BUXEY-SOFTLEY
Alan John CANTY
Maria Ann CHAMBERS
Vivienne CLEMSON
Amanda Hilary CLOUGH
Richard Derek DALE
Paul Anthony DAVIES
Ralph Paul DAY
Hugh DEVLIN
Lynn Margaret DOGGETT
Geoffrey Conrad DOWNER
Peter Maxwell DUKE
William John Hannay FALCONER HALL
Jane Helen FERGUSON
Lorraine Mary FERGUSON
Derek FIELDHOUSE
Claire Joan FOTHERINGHAM
Peter David GRIME
Patricia Mary Eizabeth HARBOURN
David John HARDY
Sophie Anne Mary HEPENSTAL
James HEROLD
Pratibha HINDOCHA
Terence Robert HITCH
Albert Ronald JUKES
Anne Patricia KEEP
Philip John KEY
Francis Neil McDONALD
Jacqueline Harriet MASTERSON
Louise Mary NASH
Carol Ann NOWILL
Simon Charles O'SHAUGHNESSY
Diana Rosalie OSTICK
Maya PATEL
Andrew Paul PRITCHARD
Judith Marion PYM
Susan Elizabeth REED
David Andrew REEKIE
Richard William RYCRAFT
Peter Charles SAWYER
John SIMPSON
Richard Charles THOMPSON
Roselyn Elizabeth TRITTON
Stephen William WHITE

**FINAL EXAMINATION FOR THE DEGREE OF B.D.S. (SECTION II) DECEMBER 1974**

**PASS**

Charles Edward COE
Geir Atle KVAMME
Patricia MUSGROVE
Carolyn Voirrey SHIMMIN

**FINAL EXAMINATION FOR THE DEGREE OF B.D.S. (SECTION III) DECEMBER 1974**

**WITH HONOURS**

Louise BENSON
Charles Marius SCOLA—with Distinction in Advanced Dental Surgery
Shirley Margaret SCOLA

**PASS**

David Maxwell ADLAM
Michael Roy BRADLEY
Michael John COAKLEY
Judith Ann COX
Angela Mary CROPPER
Celie Jane DARLINGTON
Michael John FLEETWOOD
Kent Aloysius GLACE
Robert GOODRUM
Ronald GRIBBIN
Terence Michael JONES
Willy McLean KABAMBE
Salim Gulamhusein LADHA
Robert Adam LENKO
Yee Sui LEUNG
Michael Kwok-Wang LI
Nicholas MALPELI
Per Alf MATHIESEN
Andrea MILLS
Jeremy Ronald NASH
Ian James PARK
Patricia Helen PENNINGTON
Robin PIERCE-WILLIAMS
Stephen Davies PRITCHARD
Janet REYNOLDS
David Michael ROBINSON
Robert Andrew SALVIN
Ralph-Peter SMITH
Prudence Mary WHITE
Stephen Charles WILKINSON
Paul Anthony WILLIAMS
Anthony Hugh WOOTTON
Anne Helena WYSOCKA

**EXAMINATION FOR THE CERTIFICATE IN HEALTH VISITING (PART II) 1974**

The undermentioned candidate having successfully completed her practical work is now fully qualified for the award of the Certificate in Health Visiting.

Vera Ann HEIJBOER

**FACULTY OF MEDICINE**
**THE DEGREE OF MASTER OF SCIENCE IN MEDICAL SCIENCES**
**JANUARY 1975**
**DISSERTATIONS APPROVED**

Patricia Ann BRADLEY
Morakot SUKCHOTIRATANA

**FACULTY OF MEDICINE**
**THE DEGREE OF MASTER OF SURGERY**
**JANUARY 1975**
**DISSERTATION APPROVED**

Michael John TORRENS

**FACULTY OF MEDICINE**
**THE DEGREE OF DOCTOR OF PHILOSOPHY**
**JANUARY 1975**
**DISSERTATIONS APPROVED**

Helen Kay BEARD
Barry JONES

**FACULTY OF MEDICINE**
**THE DEGREE OF DOCTOR OF MEDICINE**
**JANUARY 1975**
**DISSERTATIONS APPROVED**

Carol Mary BLACK
Arumugam GANENDRAN
Peter Roger GOLDING
Euan Macdonald ROSS

**FACULTY OF MEDICINE**
**THE DEGREE OF DOCTOR OF PHILOSOPHY**
**MARCH 1975**
**DISSERTATIONS APPROVED**

Timothy Joseph DAVID
Ratnasingham NADARAJA

### The Medical Library

In 1890 the Bristol Medico-Chirurgical Society founded a medical library which in 1892 was combined with that of the Bristol Medical School. This became the University of Bristol Medical Library in 1925 and an agreement in that year confirmed the privilege of Members of the Society to use the University Medical Library.

### A Selection of Recent Additions to the Medical Library

BELLET, S.: Clinical disorders of the heart beat. Ed. 3. (Lea & Febiger)
BOGOCH, A. ed.: Gastroenterology. (McGraw-Hill)
BOYLE, A. C.: A colour atlas of rheumatology. (Wolfe)
BRUCH, H.: Eating disorders: obesity, anorexia nervosa and the person within. (Routledge)
BULL, T. R.: A colour atlas of ENT diagnosis. (Wolfe)
BURNET, M.: Intrinsic mutagenesis: a genetic approach to ageing. (Med. Tech. Publ. Co.)
CALNE, D. B.: Progress in the treatment of Parkinsonism. (Advances in Neurology, 3) (Raven Pr.)
COMLINE, K. S. et al. eds.: Foetal and neonatal physiology. (Cambridge Univ. Pr.)
CONNOLLY, K. & J. BRUNNER: The growth of competence. (Acad. Pr.)
CRANLEY, J. J.: Vascular surgery. Vol. 1: Peripheral arterial diseases. (Harper & Row)
CREAMER, B.: The small intestine. (Heinemann)
CURRAN, R. C. & E. L. JONES: Gross pathology: a colour atlas. (Harvey Miller & Medcalf)
DIMITROV, N. V. & J. H. NODINE, eds.: Drugs and hematologic reactions. (Grune & Stratton)
DOUGLAS, C. P. & K. S. HOLT: Mental retardation: prenatal diagnosis and infant assessment (Butterworths)
FFRENCH: D. E.: Occupational health. (Med. Tech. Pub. Co.)
GAUVAIN, S. ed.: Occupational health: a guide to sources of information. (Heinemann)
GHALIOUNGUI, P.: The house of life: magic and medical science in ancient Egypt. (B. M. Israel)
GRUMBACH, M. M. et al: Control of the onset of puberty. (Wiley)
HARRIS, L.: Geriatric chest disease. (Wright) Presented by John Wright & Sons Ltd.
HOEPRICH, P.: Infectious diseases. (Harper & Row)
HOLMES, R. L. & J. N. BALL: The pituitary gland: a comparative account. (Cambridge Univ. Pr.)
HOWE, G. M. & J. A. LORAINE: Environmental medicine. (Heinemann)
HUNTSMAN, R. G. & G. C. JENKINS: Advanced haematology. (Butterworths)
JABLONSKI, S. R.: Illustrated dictionary of eponymic syndromes and diseases. (Saunders)
JANOVSKI, N. A. & C. DOUGLAS: Diseases of the vulva. (Harper & Row)
JOHNSON, R. H. & J. M. K. SPALDING: Disorders of the autonomic nervous system. (Blackwell)
KNIGHT, V. ed.: Viral and mycoplasmal infections of the respiratory tract. (Lea & Febiger)
LEE, H. A.: Parenteral nutrition in acute metabolic illness. (Acad. Pr.)
LINDENBERG, R.: Neuropathology of vision: an atlas. (Lea & Febiger)
LOSOWSKY, M. S.: Malabsorption in clinical practice. (Churchill Livingstone)
MASON, E. E.: Fluid, electrolyte and nutrient therapy in surgery. (Lea & Febiger)
MELLOR, W. F. ed.: Casualties and related statistics. (History of the Second World War; United Kingdom medical series) (H.M.S.O.)
MITCHELL, R. S.: Synopsis of clinical pulmonary disease. (Mosby)
NGAN, H. & K. W. JAMES: Clinical radiology of the lymphomas. (Butterworths)
NICHOLSON, J. P. ed.: Interdisciplinary investigation of the brain. (Plenum Pr.)
NILSSON, I. M.: Haemorrhagic and thrombotic diseases. (Wiley)
OTCENASEK, M. & J. DVORAK: Pictorial dictionary of medical mycology. (Junk)
PALLIS, C. A. & P. D. LEWIS: Neurology of gastrointestinal disease. (Saunders)
PASTEELS, J. L. & C. ROBYN, eds.: Human prolactin. (Excerpta Medica)
PAYNE, E. E.: An atlas of pathology of the brain. (Lewis)
PEARCE J. & E. MILLER: Clinical aspects of dementia. (Baillière)
PETRE-QUADENS, O. J. & J. D. SCHLAG: Basic sleep mechanisms. (Acad. Pr.)
PRIER, J. E. & H. FRIEDMAN: Opportunistic pathogens. (Univ. Park Pr.)
SCHWARTZ, S. I. et al.: Principles of surgery. Ed. 2. (McGraw-Hill)
SEVITT, S.: Reactions to injury and burns. (Heinemann)
SHERBET, G. V.: Neoplasia and cell differentiation. (Karger)
SHIELDS, T. W. et al. eds.: General thoracic surgery. (Lea & Febiger)
SHIRES, G. T.: Shock (Major problems in clinical surgery). (Saunders)
SMITH, R. E. & W. G. WILLIAMS: Surgery of the lung (Butterworths)
SNYDERMAN, R. K. ed.: Symposium on neoplastic and reconstructive problems of the female breast. (Mosby)
STEVENSON, R. E.: The fetus and newly-born infant: influence of the prenatal environment. (Mosby)
SUNDERLAND, S.: Nerves and nerve injuries. (Churchill Livingstone)
SZEKELEY, P. & L. SNAITH: Heart disease and pregnancy. (Churchill Livingstone)
TRONZO, R. G.: Surgery of the hip joint (Lea & Febiger)
VILLEE, C. A. et al: Respiratory distress syndrome. (Acad. Pr.)
WESENBERG, R. L.: The newborn chest. (Harper & Row)
WEST, J.: Respiratory physiology. (Blackwell)

